# The incredible ULKs

**DOI:** 10.1186/1478-811X-10-7

**Published:** 2012-03-13

**Authors:** Sebastian Alers, Antje S Löffler, Sebastian Wesselborg, Björn Stork

**Affiliations:** 1 Department of Internal Medicine I, University Hospital of Tübingen, Otfried-M&#252ller-Str. 10, 72076 Tübingen, Germany; 2 Institute of Molecular Medicine, University Hospital of Düsseldorf, Universit&#228tsstr. 1, Building 23.12, 40225 Düsseldorf, Germany

**Keywords:** Atg1, Atg13, Atg17, UNC-51, EPG-1, Ulk1, Ulk2, FIP200, Atg101, Autophagy, Serine/threonine phosphorylation

## Abstract

Macroautophagy (commonly abbreviated as autophagy) is an evolutionary conserved lysosome-directed vesicular trafficking pathway in eukaryotic cells that mediates the lysosomal degradation of intracellular components. The cytoplasmic cargo is initially enclosed by a specific double membrane vesicle, termed the autophagosome. By this means, autophagy either helps to remove damaged organelles, long-lived proteins and protein aggregates, or serves as a recycling mechanism for molecular building blocks. Autophagy was once invented by unicellular organisms to compensate the fluctuating external supply of nutrients. In higher eukaryotes, it is strongly enhanced under various stress conditions, such as nutrient and growth factor deprivation or DNA damage. The serine/threonine kinase Atg1 was the first identified autophagy-related gene (ATG) product in yeast. The corresponding nematode homolog UNC-51, however, has additional neuronal functions. Vertebrate genomes finally encode five closely related kinases, of which UNC-51-like kinase 1 (Ulk1) and Ulk2 are both involved in the regulation of autophagy and further neuron-specific vesicular trafficking processes. This review will mainly focus on the vertebrate Ulk1/2-Atg13-FIP200 protein complex, its function in autophagy initiation, its evolutionary descent from the yeast Atg1-Atg13-Atg17 complex, as well as the additional non-autophagic functions of its components. Since the rapid nutrient- and stress-dependent cellular responses are mainly mediated by serine/threonine phosphorylation, it will summarize our current knowledge about the relevant upstream signaling pathways and the altering phosphorylation status within this complex during autophagy induction.

## What is autophagy and how is it induced?

Although the term autophagy refers to several quite different cellular processes, a common feature of each is the final degradation of intracellular components by the lysosome (for a detailed overview of the diverse types and subtypes of molecular self-eating see [1]). Microautophagy, the "lesser-known" form of autophagy [2], refers to the direct engulfment of cytoplasm via the lysosome in mammals (or the vacuole in plants and fungi) by invagination of the lysosomal membrane. In chaperone-mediated autophagy (CMA), single proteins are selectively bound by hsc70 and transported into the lysosomal lumen. The best known and most frequently studied form of autophagy, however, is macroautophagy. Here, the cytosolic components are enclosed by a specific double membrane vesicle, termed the autophagosome. Macroautophagy hence represents the vesicular mode of transport for intracellular components to the lysosome. Since it is common practice in scientific literature to simply refer to macroautophagy as autophagy, this designation will be applied throughout the manuscript.

Autophagy can further be classified, based on the identity of the engulfed material and the purpose for which it has been degraded. Initially, autophagy has been regarded as a predominantly unspecific and random process, merely comprising the bulk degradation of cytoplasm. Meanwhile, several forms of cargo-specific sequestration have been identified [1]. This includes the autophagic degradation of mitochondria (Mitophagy), peroxisomes (Pexophagy), endoplasmic reticulum (Reticulophagy), ribosomes (Ribophagy), and protein aggregates (Aggrephagy). The term autophagy was even extended beyond its original meaning, in the sense of "self-eating", in order to cover the autophagosomal degradation of invaded or phagocytosed components such as bacteria and viruses (Xenophagy). In the above mentioned cases, autophagy either serves as a quality control and rescue mechanism - in order to get rid of the superfluous, damaged or otherwise harmful cytoplasmic constituents - or as a recycling mechanism, in order to reuse the precious molecular building blocks, especially in times of nutrient starvation. Basal autophagy hence occurs to a limited extent in nearly every eukaryotic cell. However, autophagy is remarkably enhanced under diverse cellular stress conditions, such as the deprivation of nutrients, growth factors and oxygen, the damage of DNA and mitochondria, the infection with intracellular pathogens, and the accumulation of protein aggregates (reviewed in [3]). The extent and specificity of autophagosomal degradation is hence tightly controlled by a dense signaling network that integrates the relevant information about the cellular nutrient and energy status in order to appropriately regulate the autophagic machinery.

Initially, genetic studies in yeast uncovered the existence of several so-called autophagy-related genes (ATG); by now the number has risen to 35. Their products constitute at least six functionally distinct modules: the Atg1-Atg13-Atg17 kinase complex, the Vps34-Vps15-Atg6-Atg14 class III phosphatidylinositol 3-kinase (PI3K) complex, the PI(3)P binding Atg2-Atg18 complex, the multi-spanning transmembrane protein Atg9 and the two ubiquitin-like conjugation systems Atg8-PE and Atg12-Atg5-Atg16 [4,5]. In yeast, these protein complexes are recruited in a hierarchical manner to the single site of autophagosome biogenesis, the pre-autophagosomal structure (PAS). By this means, they mainly regulate the initial steps of autophagosome formation, such as nucleation, expansion and final closure [4].

Although originally invented by unicellular eukaryotes, which live under fluctuating nutrient supply, autophagy has been adapted to the growing demands of multicellular organisms during evolution. While the molecular core machinery itself is remarkably conserved, it has been modified in several ways to account for the higher complexity and cellular diversity of higher eukaryotes. This includes the existence of multiple isoforms of several autophagy related genes (Atg1, Atg2, Atg4, Atg8, Atg9, Atg16 and Atg18), the interconnection with multiple stress-related and developmental pathways (growth-factor regulated nutrient uptake and metabolism, cell cycle, cell growth, cell survival, and cell death), as well as the overlap with other vesicular trafficking processes (endocytosis and phagocytosis). Furthermore, it became apparent that several yeast ATG gene products possess no obvious homolog in higher eukaryotes (e.g. Atg11, Atg17, Atg29 and Atg31), that other vertebrate proteins have adopted the function of some of these missing proteins (e.g. FIP200), and finally that some vertebrate Atg homologs have gained additional non-autophagy related functions during evolution (e.g. Atg1 and Atg8).

This review will mainly focus on the vertebrate Ulk1/2-Atg13-FIP200 complex, its function in autophagy initiation, its evolutionary descent from the yeast Atg1-Atg13-Atg17 complex, as well as the additional non-autophagic functions of its components. Since the rapid nutrient- and stress-dependent cellular responses are mainly mediated by serine/threonine phosphorylation, it will additionally summarize our current knowledge of the altering phosphorylation status within this complex during autophagy initiation.

## Atg1 - the one and only kinase

**Figure 1:**
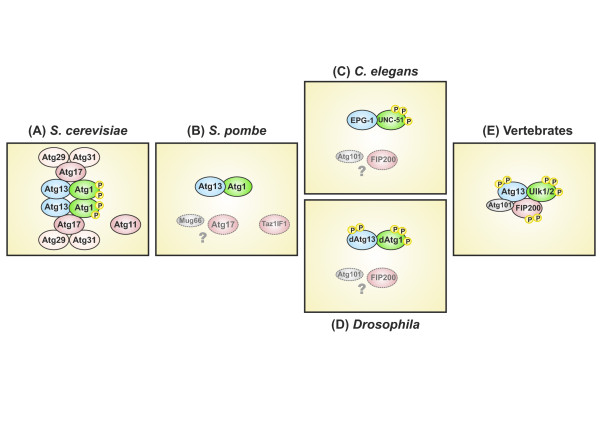
**Evolutionary descent of the vertebrate Ulk1/2-Atg13-FIP200 complex**. (**A**) In the "baker's yeast" species *Saccharomyces cerevisiae*, the protein kinase Atg1 is found in a large protein complex that comprises Atg13 and Atg17-Atg29-Atg31, once autophagy is initiated. Under starvation conditions, the hypophosphorylated protein Atg13 induces self-association of Atg1, which strongly enhances its kinase activity. *S. cerevisiae *additionally expresses Atg11, a scaffolding protein that is involved in the fungi-specific Cvt pathway. (**B**) The closely related "fission yeast" species *Saccharomyces pombe *expresses Atg1 and Atg13. Both proteins are essential for autophagy induction [79]. It additionally possesses a homolog of yeast Atg17 and a putative homolog of yeast Atg11 (Taz1IF1) [51,78]. However, Taz1IF1 shows great similarity to vertebrate FIP200 [51]. The protein Mug66 has been assigned as a putative homolog of vertebrate Atg101 [51]. The molecular details of autophagy induction have not been addressed yet. (**C**) The nematode *Caenorhabditis elegans *expresses an Atg1 homolog (UNC-51) and an interacting Atg13 homolog (EPG-1) that both are essential for autophagy induction [24,32], while the phosphorylation of EPG-1 by UNC-51 has not been determined yet. The nematode genome contains a homolog of both FIP200 (T08A9.1; assigned as *atg-11*) and Atg101 (Y69A2AR.7); their role in autophagy has not been addressed. (**D**) In the fruit fly species *Drosophila melanogaster*, dAtg1 binds and phosphorylates dAtg13. In contrast to yeast, the dAtg1-dependent phosphorylation of Atg13 is greatest under autophagic condition. The composition of the dAtg1-dAtg13 complex is not affected by the nutrient status [34,35,37]. The *Drosophila *genome contains a FIP200 homolog (CG1347) and an Atg101 homolog (CG7053). The involvement of the respective gene products has not been addressed. (**E**) Vertebrate species possess a large protein complex, comprising Ulk1 or Ulk2, Atg13, FIP200 and Atg101, whose composition is unaffected by the nutrient status [53-56,68,75,76,85]. In mammals, mTORC1 associates with this complex under normal growth conditions and phosphorylates Ulk1/2 and Atg13, thereby inhibiting Ulk1/2 kinase activity [55]. Active Ulk1/2 autophosphorylates and is able to phosphorylate both Atg13 and FIP200, but the relevance for autophagy induction has not been determined yet.

The apg1 strain was the first known autophagy-defective mutant of *Saccharomyces cerevisiae*, initially identified in a global screen for autophagy loss-of-function strains [6]. The respective gene was found to encode a serine/threonine protein kinase, subsequently termed Atg1 (originally known as Apg1) [7,8]. It still remains the only known kinase among the Atg proteins. During the following years it became obvious that the Atg1 kinase directly or indirectly interacts with numerous other ATG gene products, of which Atg13 [7], Atg17 [9,10], Atg29 [11] and Atg31 [12] are involved in the regulation of canonical macroautophagy. The current data suggest that Atg17 constitutively associates with Atg29 and Atg31 and mainly represents a scaffold that organizes the subsequent recruitment of the other Atg proteins to the PAS after autophagy initiation [13,14], while the dynamic interaction between Atg1 and Atg17 seems to be primarily mediated by Atg13 [10,15] (Figure 1). In a landmark paper, Kamada et al. demonstrated firstly, that Atg1 kinase activity is strongly enhanced during starvation; secondly, that both Atg13 and Atg17 are essential for this activity; and thirdly; that both starvation and rapamycin treatment leads to a considerable dephosphorylation of Atg13, which subsequently results in an enhanced affinity for Atg1 [9]. It was already known that the inhibition of the serine/threonine protein kinase target of rapamycin (TOR) induces autophagy in yeast, even under nutrient rich conditions, and that TOR acts upstream of Atg1 [16]. However, the observation by Kamada et al. directly links the inactivation of TOR to the activation of Atg1 kinase activity and in turn to autophagy initiation. Notably, in yeast, autophagy can be at least partially induced merely by overexpression of an Atg13 mutant, which is non-phosphorylatable by TOR [17]. The activation of Atg1, as observed after starvation, is hence primarily mediated by the dephosphorylation of several TOR-dependent phosphorylation sites in Atg13 [17] and recent data suggest that this activation is a direct result of the subsequent Atg13-mediated dimerization of Atg1 [18] (Figure 1).

In summary, the formation of the Atg1-Atg13-Atg17 complex, its recruitment to the PAS, and the subsequent enhancement of Atg1 kinase activity are followed by the recruitment of further Atg proteins to the PAS. This finally leads to the formation of autophagic vesicles in yeast [4,19]. However, it has to be pointed out that, although the kinase activity of Atg1 seems to be essential for the proper formation of functional and normally sized autophagosomes in yeast, it might be dispensable for the initial recruitment of the other modules mentioned above, such as the PI3K class III complex, the Atg2-Atg18 complex, the two ubiquitin-like conjugation systems, and Atg9 [4,14,19,20]. As previously suggested by Chan and Tooze [21], this argues for a kinase-independent function of Atg1 in the initial organization of the PAS formation, followed by the kinase-dependent function in the dynamical phase of autophagosome development. Notably, although several putative Atg1 *in vitro *substrates could be identified in a global proteomic analysis in yeast (among them Atg8 and Atg18) relevant *in vivo *substrates are still unknown [22]. This leaves the intriguing question open: how exactly is Atg1 kinase activity linked to autophagy induction in yeast?

## UNC-51 - the doubly talented kinase

Interestingly, Atg1 turned out to represent a close homolog of a previously identified *C. elegans *protein kinase, sharing 39.8% identity and 52.7% similarity in their N-terminal kinase domain [8]. It was initially termed UNC-51, since its loss resulted in an uncoordinated movement phenotype [23] and has been originally regarded as an essential factor for neuronal development. Its essential role for starvation-induced dauer development and the proper localization of autophagosomal marker proteins has been verified subsequently [24]. In accordance with its function in axon guidance and axon outgrowth, UNC-51 is most extensively expressed in neurons, especially in the head region of late embryos during embryonic development [23]. Further studies identified VAB-8 and UNC-14 as direct binding partners and substrates of UNC-51 [25,26], two proteins involved in the axonal trafficking of synaptic vesicles and endosomal trafficking of the axon guidance receptor UNC-5. VAB-8 is a kinesin-like molecule that is essential for the posteriorly directed migration and outgrowth of axons [27]; UNC-14 is a RUN-domain containing protein that regulates the subcellular localization of the axon guidance receptor UNC-5 [28] and that mediates the kinesin-1-dependent transport of synaptic vesicles [29]. Furthermore, LET-92, the catalytic subunit of the *C. elegans *serine/threonine protein phosphatase 2A (PP2AC), has been identified both as direct binding partner of UNC-51 and UNC-14 and as an antagonist of UNC-51 function [30].

As in yeast, the TOR homolog LET-363 was found to negatively regulate autophagy induction in *C. elegans*. However, it is unclear if and how LET-363 inhibition is mechanistically linked to UNC-51 activity [31]; even though a divergent homolog of yeast Atg13, termed EPG-1, could be identified and has been shown to directly interact with UNC-51 [32] (Figure 1). Interestingly, while the loss of *epg-1 *results in severe defects in autophagy-related processes, it does not result in an uncoordinated phenotype, as seen for *unc-51 *[32]. This strongly suggests that the neuronal function of UNC-51 is independent of the interaction with EPG-1 and the latter might hence represent an autophagy-specific interaction partner, just as VAB-8 and UNC-14 are for axon guidance and axon outgrowth.

The additional neuronal role of Atg1 homologs seems to be conserved throughout the metazoan lineage, since the corresponding *Drosophila *protein UNC-51/dAtg1 binds and phosphorylates UNC-76, a kinesin heavy chain (KHC) adaptor protein that mediates synaptic vesicle transport [33]. Both the loss of *unc-51/atg1 *and *unc-76 *results in defective axonal vesicular trafficking processes [33]. In addition, as observed in *S. cerevisiae *and *C. elegans*, the product of the single *unc-51*/*atg1 *gene has been shown to act in autophagy initiation, downstream of *Drosophila *TOR (dTOR) [34,35]. In *Drosophila*, overexpression of dAtg1 is even sufficient to induce autophagy [34]. Moreover, the ability of dAtg1 to *vice versa *inhibit dTOR signaling indicates the existence of a positive feedback loop that might help to amplify autophagy initiation once it is activated [34,36]. Mechanistic insights into the dTOR-dependent regulation of dAtg1 came from studies by Chang and Neufeld [37,38]. The authors could identify a weakly conserved *Drosophila *homolog of yeast Atg13 that directly interacts with dAtg1 *in vivo*. dTOR associates with the dAtg1-dAtg13 complex and both dAtg1 and dAtg13 are phosphorylated in a nutrient-dependent manner by dTOR. However, in contrast to the situation in yeast, the phosphorylation status of dAtg13 is highest when autophagy is induced - presumably via enhanced dAtg1-dependent phosphorylation - and it does not affect the composition of the dAtg1-dAtg13 complex [37] (Figure 1).

**Figure 2:**
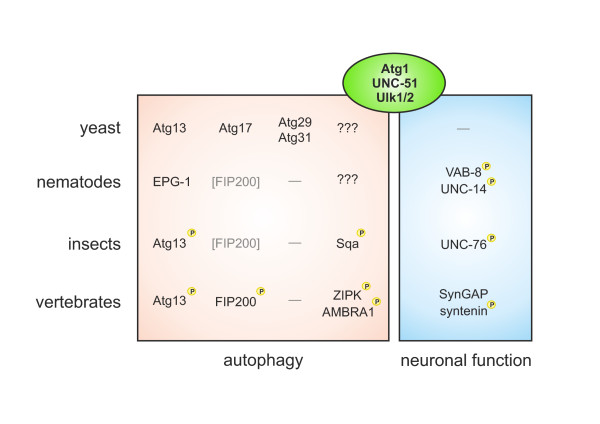
**Interaction partners and substrates of Atg1, UNC-51 and Ulk1/2**. In yeast, Atg1 directly interacts with Atg13 and Atg17-Atg29-Atg31; no autophagy-specific *in vivo *substrate of Atg1 has been identified yet. In nematodes, UNC-51 directly interacts with EPG-1; no autophagy-specific UNC-51 substrate is known. However, UNC-51 phosphorylates VAB-8 and UNC-14, two proteins involved in axonal trafficking of synaptic vesicles [25,26,28]. In insects, UNC-51/Atg1 binds and phosphorylates both Atg13 and the kinesin heavy chain adaptor protein UNC-76 that mediates synaptic vesicle transport [33]. In addition, the myosin light chain kinase named Sqa has been identified as autophagy-relevant UNC-51/Atg1 substrate [131]. In vertebrates, the UNC-51-like kinases 1 (Ulk1) and Ulk2 both directly interact with Atg13 and indirectly with FIP200 (a functional homolog of yeast Atg17). Ulk1/2 are able to phosphorylate Atg13 and FIP200, but the relevance for autophagy induction has not been determined yet. However, the Ulk1-dependent phosphorylation of Atg13 (at S318) does seem to be relevant for mitophagy [64]. The mammalian kinase ZIPK (a homolog of *Drosophila *Sqa), and the Beclin 1-interacting protein AMBRA1 have been identified as autophagy-relevant substrates of Ulk1 [131]. The synaptic proteins SynGAP and syntenin are known as neuron-specific interaction partners of Ulk1 [65], and syntenin-1 is directly phosphorylated by Ulk1 [66].

This indicates that the single Atg1 gene in worms and flies additionally regulates neuron-specific vesicular transport processes, while in yeast it is exclusively involved in vacuole-directed trafficking, such as macroautophagy and the cytoplasm-to-vacuole (Cvt) targeting pathway. The neuronal specificity depends on the interaction of (UNC-51/Atg1) with VAB-8, UNC-14 and UNC-76, respectively; in contrast to its interaction with (EPG-1/Atg13) in the case of autophagy (Figure 2). Interestingly, while in yeast the Atg1-Atg13 complex is accompanied by other essential components such as Atg17, Atg29 and Atg31, primary sequence homologs of these proteins are absent in higher eukaryotes [39].

## The incredible UNC-51-like kinases

Vertebrates have extended their autophagic toolbox even further, since they possess multiple isoforms of several autophagy related genes. Among these multiplied gene products are the protein kinase Atg1 and the ubiquitin-like molecule Atg8. The latter is covalently attached to the integral membrane lipid phosphatidylethanolamine (PE) during autophagy induction [40,41]. The lipidated Atg8-PE then localizes both to pre-autophagosomal structures and mature autophagosomes. Hence, it is commonly used as an autophagosomal marker protein. The vertebrate Atg8 gene family comprises six members: the microtubule-associated protein 1 light chain 3 A (MAP1LC3A, or short LC3A), LC3B and LC3C, as well as the GABA_A _receptor-associated protein (GABARAP), GABARAP-like 1 (GABARAPL1) and GABARAPL2 (also known as GATE-16). The LC3 and GABARAP subfamilies are both essential for autophagy initiation [42], but they act at different stages of autophagosome biogenesis. While LC3 family members are involved in elongation of the pre-autophagosomal membrane, the GABARAP proteins participate in later stages of autophagosomal maturation [43].

In addition, vertebrates possess at least five serine/threonine protein kinases in their genome that display a considerable homology to Atg1/UNC-51 in their kinase domain [21]. The first identified mammalian homologs were the two most closely related UNC-51-like kinases 1 (Ulk1) and Ulk2 [44-47]. Human Ulk1, for example, possesses an overall similarity of 41% to UNC-51 and a similarity of 29% to Atg1 [44]. In contrast to the other Atg1/UNC-51-related kinases Ulk3, Ulk4, and STK36, the similarity between Ulk1 and Ulk2 is not restricted to the N-terminal catalytic domain but comprises the entire protein, including the central proline/serine-rich (PS) and C-terminal domain (CTD) [21,48-51]. Notably, *ulk3 *mRNA was found to be up-regulated in fibroblasts after Ras-induced senescence, and overexpression of the Ulk3 protein was able to induce both autophagy and senescence in the human fibroblast cell line IMR90 [52]. However, via their highly conserved CTD, only Ulk1 and Ulk2 are able to interact with the vertebrate homolog of Atg13 [53-56], which was long thought to be absent from higher eukaryotes. After an *in silico *analysis of the non-redundant NCBI protein database, a human protein with the accession number AAH02378 (a putative product of the KIAA0652 gene) was originally assigned as a potential mammalian Atg13 homolog by Meijer et al. [39]. Chan et al. were the first who verified the interaction of Atg13 with both Ulk1 and Ulk2 and its essential role in autophagy induction [53]. The same group initially characterized the involvement of Ulk1 in autophagy initiation and in the redistribution of mammalian Atg9 (mAtg9) from the trans-Golgi network (TGN) to LC3-positive structures [57]. In addition, they previously proposed human Ulk1 as the major regulator of autophagy induction - despite their close sequence similarity and similar expression pattern - since only the knockdown of Ulk1, but not that of Ulk2, strongly reduced starvation-induced autophagy in HEK293 cells [53,58]. Notably, while UNC-51 is predominantly detected in the nervous system of *C. elegans*, Ulk1 and Ulk2 are likewise ubiquitously expressed in nearly all adult mammalian tissues [44,46,47]. Red blood cells, however, represent a notable exception, since only *ulk1 *mRNA is significantly up-regulated during terminal erythroid maturation [59]. Accordingly, *ulk1*^-/- ^mice display an impaired and delayed mitochondrial clearance in reticulocytes during red blood cell development. In addition, they display an increased mitochondrial mass in embryonic fibroblasts [59]. Notably, *ulk1*^-/- ^mice are nevertheless born viable and do not display any severe impairment of starvation induced autophagy [59], which is in clear contrast to other autophagy-specific knockout mice such as *atg5^-/- ^*and *atg7*^-/- ^[60,61]. The *ulk2*^-/- ^mice are likewise born viable, without any obvious autophagic phenotype [62,63]. This strongly suggests that Ulk1 and Ulk2 do have partially redundant functions in non-selective autophagy and that Ulk2 (or one of the other homologs) is able to compensate the loss of Ulk1, while Ulk1 is selectively involved in mitophagy. The specific involvement of Ulk1 in the selective engulfment of depolarized mitochondria, through phosphorylation of Atg13 at S318, has been recently confirmed [64]. Furthermore, the phenotype of recently generated *ulk1*^-/-^*ulk2*^-/- ^mice does support the view of a redundant function of both proteins in starvation induced autophagy, as it will be described in more detail below.

Consistent with the observations in *C. elegans *and *Drosophila*, both Ulk1 and Ulk2 possess an additional neuron-specific function. Ulk1 is essential for axon formation and neurite extension in cerebellar granule neurons (CGN) [45,65], and Ulk1/Ulk2 double-deficient CGN have been reported to display shorter axons [63]. In addition, Ulk1 interacts with both SynGAP [65], a GTPase-activating protein involved in synapse function, and syntenin, a PDZ-domain containing scaffolding protein for several synaptic proteins [65]. Furthermore, syntenin-1 has been recently identified as an Ulk1 substrate [66] (Figure 2). Both proteins are known to regulate Rab5-mediated neuronal endocytic pathways. Furthermore, the knockdown of Ulk1 and/or Ulk2 leads to shortened axons and increased numbers of axonal branches in embryonic sensory neurons, which is due to impaired endocytosis of nerve growth factor (NGF) and TrkA receptor trafficking [67].

Interestingly, Ulk1 and Ulk2 directly interact with several members of both the LC3 and GABARAP subfamily of mammalian Atg8 homologs [68,69]. Okazaki et al. already speculated that the interaction between UNC-51-like kinases and microtubule-associated light chain 3-related proteins might be closely related to their role in vesicular transport during axonal outgrowth [69]. In addition, autophagy is involved in the selective degradation of GABA_A_-receptors in *C. elegans *[70]. This observation might thus be attributed to the above mentioned physical interaction between Ulk1/2 and GABARAP. It may be worth to mention that *vice versa*, the involvement of neuron-specific binding partners of UNC-51 and Ulk1, such as VAB-8, UNC-14, UNC-76, SynGAP and Syntenin (Figure 2), in autophagic processes has not been directly addressed yet.

## Autophagy initiation by the Ulk1/2-Atg13-FIP200 complex

The complex that regulates the initial steps of autophagy induction in yeast comprises Atg1, Atg13 and Atg17-Atg29-Atg31 and its formation is negatively regulated by the major nutrient-sensing kinase TOR. Although both *C. elegans *and *Drosophila *possess an Atg1 homolog (UNC-51/dAtg1) as well as an Atg13 homolog (EPG-1/dAtg13), they seem to lack any primary sequence homolog of Atg17, Atg29 or Atg31. All bioinformatic approaches so far have failed to identify those genes [39]. Hara et al., however, identified the focal adhesion kinase (FAK) family interacting protein of 200 kDa (FIP200) both as an Ulk1-interacting protein and as an essential factor for the initial steps of autophagosome generation [71]. This large coiled-coil domain containing scaffold protein was initially identified as a regulator of the tumor-suppressor gene RB1 and is accordingly also known as RB1CC1 [72,73]. It is involved in diverse cellular processes and hence possesses various additional binding partners (reviewed in [74]). Thus, Hara and Mizushima already speculated that FIP200 might be the missing autophagy-specific binding partner of Ulk1 in vertebrates [71], just as SynGAP and syntenin are for the neuronal functions (Figure 2). Furthermore, based on the functional and architectural similarities, it might represent the functional homolog of yeast Atg17 in vertebrates [71]. Several simultaneously published or immediately following articles then provided compelling evidence for the existence of a large autophagy regulating Ulk-Atg13-FIP200 complex (>1 MDa), which is directly regulated by the mammalian TOR complex 1 (mTORC1) [54-56].

As stated above, both Ulk1 and Ulk2 are able to interact with Atg13 via their highly conserved C-terminal domain; while the interaction between Ulk1/2 and FIP200 is mainly mediated via Atg13 [53-56]. In contrast to the yeast Atg1-Atg13-Atg17 complex and in accordance with the Drosophila dAtg1-dAtg13 complex, the composition of the vertebrate Ulk1/2-Atg13-FIP200 complex does not dramatically vary between autophagic and non-autophagic conditions [55,68,75]. The phosphorylation status within the complex, however, does considerably change, depending on the current cellular nutrient and energy status. Under optimal growth conditions, the active mTORC1 physically interacts with the Ulk1/2-Atg13-FIP200 complex and phosphorylates Ulk1/2 and Atg13 [55]. mTOR inhibition or nutrient starvation results in a modest decrease in Atg13 and Ulk1 phosphorylation [54-56], and presumably a modest increase in Ulk1/2 kinase activity. Although the functional relevance has not been determined yet, since both FIP200 and Atg13 are direct substrates of Ulk1/2 [53-56], the Ulk1/2-dependent phosphorylation of both proteins might be a trigger for the translocation of Ulk1/2-Atg13-FIP200 to pre-autophagosomal structures and for autophagy initiation (Figures 1 and 2).

Independently, two groups identified a formerly uncharacterized protein as an additional constituent of the vertebrate Ulk1/2-Atg13-FIP200 complex [68,75,76]. This protein is encoded in the genome of worms and flies but has no obvious homolog in *Saccharomyces cerevisiae; *accordingly it was termed Atg101. It directly binds and stabilizes Atg13, most likely by preventing its proteasomal degradation [75,76].

Notably, the closely related "fission yeast" species *Saccharomyces pombe *does possess a putative Atg101 homolog that was originally termed Mug66 [75,77]. Mizushima already suggested that *S. pombe *might represent an interesting model system to study the evolution of autophagic processes [51,78,79] for the following reasons: Like *S. cerevisiae *it possesses a potential Atg17 protein and a putative Atg11 homolog (Taz1IF1), like higher eukaryotes it lacks Atg29 and Atg31 but rather has an Atg101 homolog (Mug66). However, Taz1IF1 shows a greater similarity to vertebrate FIP200 than to yeast Atg11 [51]. FIP200, on the other hand, is assigned as member of the Atg11 family in the NCBI Pfam database [71,80]. Furthermore, yeast Atg17 additionally shows a weak sequence similarity to vertebrate Atg101 [80]. In yeast, Atg17 and Atg11 both interact with Atg1 and serve as scaffolding proteins at the PAS; Atg11 under normal growth conditions as part of the cytoplasm-to-vacuole (Cvt) pathway, Atg17 under nutrient starvation as part of the autophagic machinery [19]. Thus, it is tempting to speculate that parts and function of both Atg11 and Atg17 have been transferred to other proteins such as FIP200 and Atg101 during evolution (Figure 1).

Although Ulk1/2, Atg13, FIP200, and Atg101 each could be identified as an essential factor for the initiation of autophagy and found to translocate to the site of autophagosome generation - presumably as part of a common complex - less is known about how exactly this event is linked to the initiation of autophagosome generation. In a thorough hierarchical analysis of several mammalian Atg proteins, Itakura and Mizushima identified Ulk1/2-Atg13-FIP200 as the most upstream complex in starvation induced autophagy [81], whose recruitment to the endoplasmic reticulum (ER) is essential for the recruitment of further autophagy-related protein complexes, such as the Vps34-Vps15-Beclin 1(Atg6)-Atg14L complex. The catalytic activity of Vps34 in turn leads to recruitment of PI(3)P-binding proteins (WIPI-1/2 and DFCP1) and subsequently to the recruitment of both the LC3 and the Atg12-Atg5-Atg16L1 conjugation system. However, this hierarchy of recruitment in general and the role of Ulk1/2-Atg13-FIP200 in particular, again seem to depend on the stimulus of autophagy induction. For example, for *Salmonella *xenophagy it could be recently shown that Ulk1 is required for the cycling of Atg9L1 (Atg9A) and Atg14L towards an autophagosome-like double-membrane structure which surrounds *Salmonella*-containing vacuoles. In contrast, the recruitment of the LC3 conjugation system to these structures occurred independently of Ulk1 [82]. In recent years, a new group of autophagic adaptors has been identified, which recognize intracellular pathogens and target them for autophagic degradation [83]. These p62/sequestasome-like receptors (SLRs) are part of the innate immune defense and contain an LC3-interacting region (LIR) combined with an ubiquitin-binding region. By virtue of this, ubiquitinated pathogens are connected to nascent LC3-positive autophagic membranes [83]. Future studies will have to reveal how Ulk1 (and Ulk2) contribute to the function of autophagic adaptors. Given the evolutionary descent of mitochondria from bacterial symbionts, one might again learn some lessons from mitophagy. It has been suggested that Parkin-mediated ubiquitination of the mitochondrial proteins VDAC1 and mitofusin targets depolarized mitochondria for autophagic degradation [83]. The selective involvement of Ulk1 in mitochondrial clearance has been described above. Furthermore, Mizushima's group could recently show that the Ulk1 complex and Atg9A are independently recruited to depolarized mitochondria and are both required for further recruitment of downstream Atg proteins, except LC3 [84].

The conception of Ulk1/2-Atg13-FIP200 as a functional unit is, however, mainly derived from the complete autophagy-defective phenotype of *fip200*^-/- ^MEFs [81,85], as well as the fact that FIP200 is a direct or indirect binding partner of Atg13, Ulk1, Ulk2 and Atg101, that all five proteins can be found in a common high-molecular-weight-complex, and that each of these proteins translocates to pre-autophagosomal structures after autophagy induction [53-56,68,75,76,85]. The single knockdown of individual components, however, has remarkably differing effects on autophagy initiation [53-56,63,68,75,76,85]. A deeper insight into the functional hierarchy within this complex will hence crucially depend on the existence of appropriate vertebrate knockout models and on the expansion of the above mentioned hierarchical analysis to these systems. The autophagic phenotype of *atg13*^-/- ^and *atg101*^-/- ^mice is hitherto unpublished. As described above, the single *ulk1*^-/- ^and *ulk2*^-/- ^mice are both born viable and do not display a general autophagy-defective phenotype [59,62,63]. The double-knockout of Ulk1 and Ulk2, in contrast, leads to neonatal lethality. Furthermore, embryonic fibroblasts from *ulk1^-/-^ulk2*^-/- ^mice are completely unresponsive to amino acid starvation [62]. This argues for a functional redundancy and an essential function of both proteins in starvation induced autophagy, at least in response to amino acid withdrawal.

Collectively, it will be crucial to distinguish between the functions that proteins fulfill dependent and independent of the Ulk1/2-Atg13-FIP200 complex. Dissecting the redundancy between Ulk1 and Ulk2 and between their autophagic- and non-autophagic (neuronal) functions will be in particular a challenging task. FIP200, in addition, is a multi-functional protein that is not only involved in autophagy but also in cell growth, proliferation and survival, as well as in cell adhesion and migration [74].

Although the Ulk1/2-Atg13-FIP200 complex is regarded as the major and most upstream factor in starvation induced autophagy, there is a growing body of evidence that autophagy can be induced even in the absence of some of its components. While *fip200*^-/- ^MEFs display a complete blockage of basal autophagy and do not respond with autophagy induction to various stimuli [85], the MEFs from *ulk1^-/-^ulk2*^-/- ^mice still respond with autophagy induction to long-term glucose starvation and increasing extracellular concentrations of ammonia [62]. Glutamine-derived ammonia has been previously identified as a diffusible factor that is able to induce autophagy [86,87]. As early as in the 1920s, Otto Warburg discovered that highly proliferative cancer cells display an abnormally changed metabolism and preferentially rely on the less effective glycolysis for ATP production [88]. Even under aerobic conditions, mitochondria do not provide ATP by oxidative phosphorylation, but provide biosynthetic precursors via the tricarboxylic acid (TCA) cycle. Since glycolysis-derived pyruvate is predominantly reduced to lactate - and glucose thus cannot be used as a carbon source to maintain the TCA cycle - rapidly growing cells mainly rely on glutamine. The mitochondrial glutaminolysis reaction, however, produces ammonia as a diffusible byproduct that acts both as an auto- and paracrine activator of autophagy [86]. By this means, long-term glucose withdrawal is able to induce autophagy, even in *ulk1^-/-^ulk2*^-/- ^MEFs [62]. However, it is still an intriguing question whether ammonia would induce autophagy in *atg13*^-/- ^or *fip200*^-/- ^cells, and *vice versa *how *ulk1^-/-^ulk2*^-/- ^and *atg13*^-/- ^MEFs would react to stimuli that failed to induce autophagy in *fip200*^-/- ^MEFs [85]. Notably, in this regard, using the vertebrate B cell line DT40, our group discovered that *ulk1^-/-^ulk2*^-/- ^cells do not show any obvious autophagy-defective phenotype and normally respond with starvation induced autophagy [89]. The respective *atg13*^-/- ^DT40 cell line, in contrast, shows a complete blockage of starvation induced and basal autophagy [89]; the same applies to *fip200*^-/- ^DT40 cells (unpublished observation). The obvious differences between *ulk1^-/-^ulk2*^-/- ^MEFs and *ulk1^-/-^ulk2*^-/- ^DT40 cells might be explained by the fact that these two systems represent different cell types, i.e. fibroblasts versus B lymphocytes. Notably, for mammalian B lymphocytes, cell type-specific functions of autophagy have been reported, including antigen presentation or the linkage between antigen receptor and co-stimulatory signaling [90,91]. Alternatively, the differences might be due to the evolutionary divergence of aves and mammalia. Ulk1/2-independent pathways are currently intensively investigated, but the mechanistic details - such as inducing stimuli, cell type dependence, and downstream signaling components - are far from being fully understood. However, it is tempting to speculate that FIP200 and Atg13 may have a more basal and yet unknown function in vertebrates, beyond their well-established role in promoting the functions of Ulk1 and Ulk2.

In addition, Nishida et al. recently reported an unconventional and less well-studied form of macroautophagy in response to cellular stress *in vitro*. This alternative form is independent of Atg5 and Atg7 - two essential components of the ubiquitin-like conjugation system - and is hence not accompanied by LC3-lipidation. Nevertheless, it seems to depend on Ulk1 and FIP200 as well as the Beclin 1 and Vps34-containing class III PI3K complex [92]. Interestingly, since the authors did not observe any defect in erythroid differentiation in *atg5*^-/- ^mice, as reported for *ulk1*^-/- ^mice [59], they have argued that this alternative form might be responsible for mitochondrial clearance *in vivo *[92].

## Upstream of the Ulk1/2-Atg13-FIP200 complex

The three major signaling nodes mTORC1, AMPK and p53 are well known to integrate several stress-related pathways and transmit them to the Ulk1/2-Atg13-FIP200 complex.

As described above, mTORC1 negatively regulates the Ulk1/2-Atg13-FIP200 complex by direct phosphorylation. The catalytic activity of mTORC1 itself is positively regulated by growth factor signaling via the class I PI3K-Akt pathway, either by inhibition of TSC1/2 [93-96] or PRAS40 [97]. Amino acids on the other hand facilitate the Rag-GTPase-dependent recruitment of mTORC1 to the lysosomal membrane, where it is subsequently activated by Rheb-GTPases [98-100].

The AMP-activated protein kinase (AMPK) is activated under decreasing ATP/AMP ratios [101] and is able to positively regulate autophagy induction [102]. This is achieved by the inhibition of mTORC1, either via the TSC1/2-Rheb pathway [103] or by direct phosphorylation of the mTORC1 component raptor [104]. Recently it has been discovered that in addition AMPK is able to phosphorylate and activate Ulk1 and Ulk2, and by this means directly regulates Ulk1/2 kinase activity [105-110]. The interaction between AMPK and Ulk1/2 on the other hand is negatively regulated by mTORC1 [107]. Finally, Ulk1/2 are able to phosphorylate and negatively regulate both their positive and negative regulators, AMPK [111] and mTORC1 [112,113]. For a more detailed summary of the intricate interplay between mTORC1, AMPK and Ulk1, including both negative feedback and feed-forward amplification loops, see [114].

The tumor suppressor protein p53 is activated by various cellular stresses like hypoxia, DNA damage, and oncogenic stress. Interestingly, p53 is both known as a negative and positive regulator of autophagy [115]. Activated p53 induces autophagy either by inhibiting mTORC1 activation via the AMPK-TSC1/2 pathway [116], most likely through transcriptional up-regulation of AMPKβ-1/2, TSC2 [117] and Sestrin1/2 [118,119], or by the up-regulation of other pro-autophagic factors such as the damage-regulated autophagy modulator (DRAM) [120]. Interestingly, Ulk1 and Ulk2 have been additionally identified as transcriptional targets of p53 upon DNA damage [121]. On the other hand, cytoplasmic p53 was found to negatively regulate autophagy in a yet unknown manner [122,123]. This cytoplasmic function, however, seems to be closely related to its ability to directly interact with FIP200, since a single mutation in p53 (K382R) abolishes both the binding to FIP200 and its anti-autophagic capacity [124]. At first sight, this schizophrenic action of p53 in autophagy regulation may appear puzzling. However, the double-edged nature of p53 with regard to cell survival has already been well-established. Low basal levels of p53 are pro-survival under normal growth conditions, while high levels of p53 have the opposite effect under severe stress conditions [115]. Thus, it has been argued that likewise, a basal level of p53 activity is mainly anti-autophagic (especially since the loss of p53 induces autophagy even under normal growth conditions), while only activated p53 is pro-autophagic, primarily under cellular stress conditions such as oncogenic or genotoxic stress [115].

As stated above, autophagy can be induced by various means, and experimental set-ups frequently target various signaling cascades simultaneously. In addition, the undersupply of nutrients such as glucose or amino acids, of growth factors and oxygen does not only target autophagy but also apoptosis and other stress-related pathways. Furthermore, these conditions will severely affect mitochondrial functions, especially after long periods of inadequate external supply and under extremely lowered cellular ATP and oxygen levels. This includes major changes in mitochondrial metabolism and membrane potential, which eventually results in an excessive production of reactive oxygen species (ROS), mitochondrial outer membrane permeability (MOMP) and the release of pro-apoptotic factors [125]. Lowered ATP levels, ROS, and the resulting DNA damages in turn are able to simultaneously induce autophagy, mitophagy and other stress related pathways that help to limit damages and to remove depolarized mitochondria [3,125,126].

In this regard, it might be worth to note that hypoxia can elicit quite different autophagic responses, depending on the cellular system and the exact nature of the respective stress conditions. In MEFs, hypoxia induces adaptive mitophagy, which may help to maintain oxygen homeostasis under prolonged hypoxic conditions [127]. In tumor cells however, hypoxia does not specifically induce the autophagic engulfment of mitochondria [128]. Thus, it has been argued that cancer cells, which largely depend on glycolysis rather than oxidative phosphorylation, might hence have a limited need for adaptive elimination of mitochondria under low oxygen levels [129].

## The heroic actions of the ULKs - downstream targets of Ulk1 and Ulk2

Ulk1 and Ulk2 are highly autophosphorylated proteins, and the overexpression of kinase-dead (KD) mutants of both proteins exhibits a dominant negative effect on Ulk1/2-regulated pathways. The phosphorylation sites within Ulk1 have first been mapped by Dorsey et al., by comparing the phosphorylation status of wild-type and KD protein [130]. Interestingly, Chan et al. observed that an Ulk1 K46R mutant retains sufficient catalytic activity to maintain the autophosphorylated status. Hence, its overexpression does not markedly inhibit autophagy initiation, which is in contrast to the respective K46I substitution [53]. However, the overexpression of Ulk1 K46R does inhibit axon outgrowth in mice [45]. Thus, Chan and Tooze have argued for a model in which the autophosphorylation of Ulk1 and Ulk2 mainly regulates their conformation, the exposure of the CTD and by this means their interaction with other proteins [21,53,58].

Atg13 and FIP200 are known to interact with Ulk1/2 in a CTD-dependent manner, and both proteins have been identified as a direct Ulk1/2 substrate [53,55,56]. However, the relevance of this phosphorylation for starvation induced autophagy is still unknown (Figures 1 and 2). Interestingly, only Ulk1 but not Ulk2 was found to directly phosphorylate Atg13 at S318 [64]. This phosphorylation leads to the selective translocation of Atg13 to depolarized mitochondria and is essential for the efficient removal of damaged organelles during mitophagy [64]. This might mechanistically explain the exclusive involvement of Ulk1 in the clearance of mitochondria.

AMPK and mTORC1 are additionally known to directly interact with the Ulk1/2-Atg13-FIP200 complex and have been identified as direct targets of Ulk1 and Ulk2 [111-113]. These phosphorylation events, however, just help to fine tune autophagy induction, either to amplify autophagy induction by maintaining mTOR inhibition or to restrict the extent of autophagy initiation by inhibiting AMPK.

In yeast and *C. elegans*, no autophagy-specific substrate has been identified so far that would allow us to establish a direct link between the activation of Atg1/UNC-51 and the activation of the autophagic machinery. In *Drosophila*, however, the myosin light chain kinase (MLCK) termed Spaghetti squash activator (Sqa) has been identified as an autophagy-relevant Atg1 substrate [131]. This mechanism seems to be conserved in vertebrates, since the respective mammalian Sqa homolog zipper interacting protein kinase (ZIPK; also known as death-associated protein kinase 3, DAPK3) plays an essential role in starvation induced autophagy (Figure 2). The subsequent MLCK-dependent activation of the actin-associated motor protein myosin II regulates the trafficking of mAtg9 from the trans-Golgi network to the site of autophagosome generation [131]. The findings by Tang et al. hence mechanistically connect the initial observation by Young et al. [57], that Ulk1 (but not Ulk2) is essential for mAtg9 redistribution, to the Ulk1-dependent activation of the actomyosin complex. However, whether Ulk2 is likewise able to phosphorylate ZIPK (thereby having a redundant function in myosin II activation) has not been directly addressed yet. Furthermore, although the multi-spanning membrane protein mAtg9 is an essential autophagy-related protein and it has been implicated in providing membranes for the nascent autophagosomes, its exact function is still unknown [132].

As described above, the Ulk1/2-Atg13-FIP200 complex has been placed most upstream of the other autophagy-related gene products. Its activation and initial recruitment to pre-autophagosomal structures (presumably at the ER) serves as a starting signal for the subsequent recruitment of the other factors, such as the class III PI3K complex [51,81]. The core PI3K complex comprises the catalytic subunit Vps34, the regulatory subunit Vps15 (also known as p150) and Beclin 1, the mammalian homolog of yeast Atg6. AMBRA1 has been identified as an additional component of the complex and as an essential factor for autophagy induction that mainly promotes the interaction between Vps34 and Beclin 1 [133]. In addition, AMBRA1 is a direct substrate of Ulk1, but once again, its phosphorylation by Ulk2 has not yet been determined [134]. Based on their observations, the authors have proposed the following model: Under normal growth conditions, the PI3K complex associates with the dynein motor complex via direct interaction between AMBRA1 and dynein light chains 1 and 2 (DLC1/2). Upon autophagy induction and subsequent Ulk1 activation, AMBRA1 is phosphorylated by Ulk1, the PI3K complex is released and subsequently translocates to the site of autophagosome generation [133,134] (Figure 2).

The exocyst is a large hetero-octameric complex that has a well-established role in tethering post-Golgi vesicles to the plasma membrane. Only recently, it has been discovered by Bodemann et al. that the exocyst might in addition provide a dynamical scaffold for the autophagic core complexes, mentioned above [135]. Under normal growth conditions, the Ulk1/2-Atg13-FIP200 and the class III PI3K complex are primarily associated with a Sec5-containing inactive exocyst complex in the perinuclear region. In response to starvation, the activated small GTPase RalB promotes the replacement of Sec5 by the alternative component Exo84. The Exo84-complex subsequently localizes to less well-characterized vesicular structures (that might represent the sites of autophagosome generation) and additionally recruits both ubiquitin-like conjugation systems. It has been argued that the Exo84-containing exocyst complex hence might bring all relevant components of the autophagic machinery into close proximity, and by this means coordinates autophagosome biogenesis in a RalB-regulated manner [135]. Collectively, it will be a demanding task to reconcile our fragmentary information about the various autophagy-related protein complexes and subcomplexes, their functional hierarchy, spatio-temporal distribution and mutual regulation. How to combine e.g. the role of the exocyst with the dynein motor complex? How to combine the notion of a hierarchical recruitment of Ulk1 and the PI3K complex with the simultaneous recruitment of both complexes via the exocyst complex? Does the exocyst redistribute to the ER, proximal to omegasomes? And finally, if and how does Ulk1 (and/or Ulk2) regulate either the activity or the distribution of the PI3K complex as well as the targeted redistribution of mAtg9? Especially the cytoskeleton has drawn growing attention in this regard. It is well conceivable that the catalytic activity of Ulk1 and Ulk2 broadly affects the dynamical reorganization of the cytoskeleton. Future studies may hence reveal further cytoskeleton-related downstream targets.

## Conclusions

The investigation of autophagy is a rapidly growing and accelerating field of research. Or as Daniel Klionsky put it, we went "from phenomenology to molecular understanding in less than a decade" [136]. The term "autophagy" was originally brought up in 1963 by the Nobel laureate Christian de Duve, who initially discovered the lysosome in 1955 and first described the characteristic double-membrane vesicles termed autophagosomes [137]. The concept of an "autophagic machinery", whose investigation once was initiated by the discovery of the autophagy-related genes in yeast [6], meanwhile turned into the idea of a "network organization of the autophagy system" [68]. Despite our impressive proceedings during this time, we are not running out of open questions and still encounter one surprise after another. Indeed, our knowledge about the molecular details is rapidly growing: additional selective forms of autophagy that have become entitled to gain a separate designation; furthermore new vertebrate-specific autophagy-related gene products have been identified, as well as more and more molecules that are additionally involved in non-autophagic cellular processes (presumably as part of different protein complexes). Among them are Ulk1 and Ulk2, FIP200 and several proteins known to be involved in both autophagy and apoptosis (e.g. Beclin 1, Bcl-2 family members, and Atg5) [138,139]. The interconnectivity of different vesicular trafficking and signaling pathways constitutes a dense network of protein interaction and mutual posttranslational modification - mainly serine/threonine phosphorylation and ubiquitination in the case of autophagy. This puzzling situation on the one hand impedes the assignment of discrete functions to individual proteins and thus hampers our deeper understanding of the molecular details; on the other hand it challenges our inquiring minds to discover more and more pieces of this constantly growing puzzle.

## Abbreviations

ATG: Autophagy-related gene; UNC-51: uncoordinated-51; Ulk1: UNC-51-like kinase 1; FIP200: focal adhesion kinase-interacting protein of 200 kDa (also known as RB1-inducible coiled-coil protein 1 [RB1CC1]); TOR: target of rapamycin; mTORC1: mammalian TOR complex 1; AMPK: AMP-activated protein kinase; AMBRA1: autophagy/beclin 1 regulator 1; MLCK: myosin light chain kinase; Sqa: Spaghetti squash activator; ZIPK: zipper interacting protein kinase (also known as death-associated protein kinase 3 [DAPK3]); PI3K: phosphatidylinositol 3-kinase; PAS: pre-autophagosomal structure; Cvt: cytoplasm-to-vacuole targeting pathway.

## Competing interests

The authors declare that they have no competing interests.

## Authors' contributions

SA drafted and wrote the manuscript. ASL, SW and BS substantially contributed to the conception and preparation of the manuscript and its critical revision. BS and SA prepared the figures. All authors approved the final version of the manuscript.
